# Knowledge, attitudes, perceptions and practices of selected commercial dairy cattle farmers regarding neosporosis in South Africa

**DOI:** 10.4102/ojvr.v93i1.2256

**Published:** 2026-03-23

**Authors:** Whatmore M. Tagwireyi, Darshana Morar-Leather, Peter Thompson, Luis Neves, Gema Alvarez-Garcia

**Affiliations:** 1 Department of Clinical Sciences, Ross University School of Veterinary Medicine, Basseterre, Saint Kitts; 2 Department of Veterinary Tropical Diseases, Faculty of Veterinary Science, University of Pretoria, Pretoria, South Africa; 3 Department of Production Animal Studies, Faculty of Veterinary Science, University of Pretoria, Pretoria, South Africa; 4 Centro de Biotecnologia, Universidade Eduardo Mondlane, Maputo, Mozambique; 5 SALUVET Group, Animal Health Department, Faculty of Veterinary Sciences, Complutense University of Madrid, Madrid, Spain

**Keywords:** KAPs, bovine neosporosis, dairy production, South Africa, abortions

## Abstract

*Neospora caninum* is a cyst-forming coccidian protozoan with a broad host range and is maintained through a complex facultative heteroxenous life cycle involving definitive canid hosts and various warm-blooded intermediate hosts. In cattle, it is a major cause of infectious abortion, leading to significant losses in productivity and profitability worldwide. This study surveyed 48 commercial dairy farmers across seven milk-producing regions in South Africa to assess knowledge, attitudes, perceptions and practices regarding bovine neosporosis. Most respondents were commercial farmers (98%), managed mixed-breed cattle (48%), operated farms larger than 500 hectares (48%) and had cattle averaging 3–5 years in age (77%). Only 21% of farmers were aware of neosporosis. Despite limited knowledge, 77% demonstrated good disease management practices, and 52% had positive attitudes towards control efforts. Three variables were significantly associated with the odds of positive practice and positive attitude scores. Farms with herds over 500 animals were 41.7 times more likely (95% confidence interval: 1.5–5237.3) to exhibit good practices. Farms that used total mixed ration were associated with better practices, while the presence of wildlife was associated with poorer attitudes. Additionally, farms that reported the presence of wildlife were less likely to have a positive attitude score. No specific factors were found to be associated with increased disease knowledge.

**Contribution:**

This study highlights the critical knowledge gap and the need for greater awareness and targeted biosecurity measures for bovine neosporosis. It also identified neosporosis as a neglected abortifacient in dairy cattle in South Africa.

## Introduction

*Neospora caninum* is a cyst-forming coccidian protozoan with a broad host range, but a preference for cattle and dogs (Almería & López-Gatius 2013; Dubey et al. 2017). It is maintained through a complex facultative heteroxenous life cycle involving definitive canid hosts and warm-blooded intermediate hosts (Dubey, Schares & Ortega-Mora 2007). Reproductive losses, including abortions and stillbirths, particularly in dairy cattle, have been reported globally and have led to substantial economic losses (Haddad, Dohoo & VanLeewen 2005; Reichel et al. 2013). Cattle can acquire infection both horizontally, through ingestion of sporulated oocysts in contaminated feed, and vertically, through transplacental transmission of tachyzoites (Dubey & Schares 2011).

Dairy farming is the fourth-largest agricultural sector in South Africa, playing a crucial role in food security. In 2023, because of increased production, the country became a net exporter of dairy products (Milk Producers’ Organisation 2023). Common causes of abortion in dairy herds in South Africa include brucellosis, coxiellosis, Rift Valley fever, chlamydiosis, bovine viral diarrhoea virus and infectious bovine rhinotracheitis. However, there is limited information on *N. caninum* infection in dairy cattle in South Africa. A few studies have reported *N. caninum* infection in cattle in South Africa with one study in dairy cattle from milk-producing provinces reporting a seroprevalence of 2.3% (Tagwireyi et al. 2024). Another study in beef cattle in Gauteng found a seroprevalence of 9% (Njiro et al. 2011), and a third study in communally grazed cattle near the Kruger National Park reported a seroprevalence of 1.6% (Adesiyun et al. 2020). Although the parasite is one of the most commonly diagnosed abortifacients worldwide (Mee 2023), it has largely been neglected or gone unrecognised in South Africa.

Obtaining information on the knowledge, attitude and practices (KAPs) of farmers on specific diseases is important for the effective planning, implementation and assessment of strategies aimed at preventing infection and controlling disease (Bayantassova et al. 2023). It helps in the identification of knowledge gaps that may hinder animal health programmes. Few studies have been published on the KAPs of farmers regarding animal health issues in cattle in South Africa (Ngoshe et al. 2022; Olaogun et al. 2023; Tempia et al. 2019). However, to the authors’ knowledge, no studies have been published specifically addressing the KAPs regarding bovine neosporosis in South Africa. There are, however, two studies on the KAPs related to *N. caninum* from elsewhere in Africa: one in dairy herds in Egypt (Gaber et al. 2021) and another in cattle and small stock in Namibia (Samkange et al. 2023). Both studies indicated a lack of knowledge among farmers regarding *N. caninum* infection. This study, which is the first of its kind in South Africa, aimed to assess the KAPs of dairy cattle farmers regarding bovine neosporosis and to have a better understanding of the measures and strategies in place to prevent the introduction and spread of the disease.

## Research methods and design

### Study area

**FIGURE 1:**
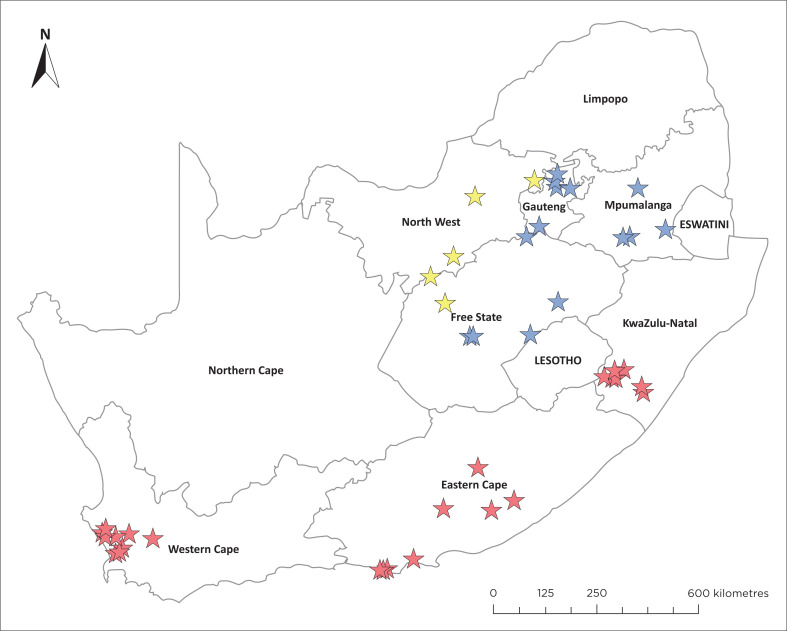
Map of South Africa highlighting three regions, with the locations of interviewed farmers marked by coloured stars: red for the southern and coastal region, blue for the central and Highveld region, and yellow for the northern and arid region.

The study was conducted across seven of South Africa’s nine provinces (Figure 1). Dairy farmers were selected from the country’s main milk-producing provinces, which together account for over 85.4% of the nation’s total milk production (Milk Producers’ Organisation 2023). South Africa spans from 22°S to 35°S and from 17°E to 33°E, with a generally temperate climate (World Bank Group 2021). The study area is divided into three regions: Southern Coastal, which includes the Western Cape, Eastern Cape and KwaZulu-Natal; Central and Highveld, which includes Free State, Mpumalanga and Gauteng; and Northern Arid, which includes the North West.

### Sample size determination and farmer selection

This study was conducted alongside a seroprevalence study of *N. caninum* (Tagwireyi et al. 2024), which determined that 1401 animals from 48 farms across seven provinces were to be sampled. The KAP survey was designed to estimate the prevalence of positive KAP scores, with a 50% positive rate and 15% precision at a 95% confidence level (CI) (Thrusfield & Robert 2018). The study was conducted between February 2022 and September 2023 using a closed-ended, paper-based questionnaire administered through face-to-face interviews as part of a seroprevalence study purposively designed to represent major milk-producing regions in South Africa. Questionnaire development involved a comprehensive lecture review on neosporosis, identification of key areas for investigation, which were formulated into questions and reviewed by three subject-matter specialists. The draft questionnaire was pretested among colleagues, and revisions were made based on the feedback received. Data on farm demographics, farmers’ knowledge of bovine neosporosis and herd management, including biosecurity, reproductive health, production parameters and the role of potential hosts of *N. caninum*, were collected. Informed consent was obtained and confidentiality maintained. Each interview took about 40 min.

### Statistical analyses

The questionnaire data were entered into Microsoft Excel^®^ (Microsoft Corporation, United States [US]) and analysed using Stata^®^ 18 (StataCorp, College Station, TX, US). Descriptive statistics were used to present the data, which were grouped into knowledge, practice and attitude categories. Each response was scored as follows: +1 for correct, 0 for unsure and −1 for incorrect. Total scores greater than 0 indicated a positive outcome in each category. Associations between potential predictors and positive knowledge, practice and attitude scores were assessed using a two-tailed Fisher’s exact test. Independent variables with *p* < 0.25 were considered for multivariable analysis. Owing to the low number of observations and occurrence of zero counts in some categories, exact logistic regression was used. Region was included in each model as part of the survey design. A forward stepwise variable selection approach was used, and variables were retained in the model if they were significant at *p* < 0.05.

### Ethical considerations

This study received ethical approval from the Faculty of Veterinary Science Research Ethics Committee and the University of Pretoria Animal Ethics Committee (REC085-20).

## Results

### Characteristics of sampled farmers and farms

A total of 48 farms were sampled. All interviewed farmers were male and operated family-run farms, with all farms located on privately owned land. Of the 48 farms, 47 were commercial farms and one was subsistence. Only three farmers had farms less than 10 hectares, while five farmers had farms between 10 hectares and 100 hectares, 17 farmers had farms between 101 hectares and 500 hectares, and 23 farmers had farms larger than 500 hectares. The number of dairy cattle on farms ranged from 12 to 11 000. Most of the farms (*n* = 32/48) were located at least 10 km away from towns and farmed predominantly mixed-breed cattle (Table 1). Most of the farms (79.1%) used computerised records, while 14.6% used handwritten records and only 6.3% had no records.

**TABLE 1:** Characteristics of sampled farms and demographics of sampled farmers.

Variable category	Response	Frequency (*n*)	Percentage (%)
Location	Southern and coastal	28	58
	Central and Highveld	15	31
	Northern and arid	5	10
Size	< 100 hectares	8	17
	101–500 hectares	17	35
	> 500 hectares	23	48
Number of cattle	< 100	8	17
	101–1000	17	35
	> 1000	23	48
Average age of cattle	< 3 years	8	17
	3–5 years	37	77
	> 5 years	3	6
Breed of cattle	Holstein Friesland	16	33
	Jersey	5	10
	Holstein–Jersey cross	4	8
	Mixed	23	47
Production system	Commercial	47	97
	Non-commercial	1	2
Proximity to town	< 5 km	4	8
	5–10 km	12	25
	> 10 km	32	66
Animal records	No recording	3	6
	Handwritten	7	15
	Computer software	38	79
Water source	Natural source	12	25
	Artificial source	24	50
	Both	12	25

### Knowledge

Generally, most farmers lacked knowledge about neosporosis (*n* = 46/48) (Table 2), with only 20.8% (*n* = 10/48) having a positive knowledge score regarding the disease. This suggests that most farmers were unaware of the specific practices needed to prevent infection in their animals.

**TABLE 2:** Knowledge of South African commercial dairy farmers (*n* = 48) regarding neosporosis-related risk factors.

Knowledge	Response	Frequency (*n*)	Percentage (%)
Knowledge of neosporosis	Yes	2	4.2
	No	46	95.8
Knowledge of the causes of abortions	Yes	11	22.9
	No	37	77.1

### Practices

A significant proportion of farmers, 77% (*n* = 37/48), exhibited a positive score for practices related to disease prevention. Notably, most farmers did not test replacement animals for any other diseases except for the mandatory brucellosis and tuberculosis tests, with 85.4% (*n* = 41/48) reporting that they did not test their replacement animals (Table 3). Furthermore, 89.6% (*n* = 43/48) of farmers reported having dogs on their properties, whether owned or stray.

**TABLE 3:** Practices of South African commercial dairy farmers (*n* = 48) towards neosporosis-related risk factors.

Practices	Response	Frequency (*n*)	Percentage (%)
Segregation of cattle by age	Yes	5	10
	No	43	89
Hygiene and sanitary conditions	Excellent	14	29
	Good	33	68
	Poor	1	2
Cattle fed total mixed ration	Yes	15	31
	No	33	69
Testing of replacement animals	Yes	7	15
	No	41	85
Cattle kraaled or housed at night	Yes	28	58
	No	20	42
Closed herd	Yes	34	71
	No	14	29
Quarantine of replacement animals	Yes	9	19
	No	39	81
Biosecurity of cattle husbandry practices	High	6	13
	Moderate	35	73
	Low	7	15
Presence of dogs on farms	Yes	43	90
	No	5	10
Dogs’ access to afterbirth or aborted foetuses	Yes	27	56
	No	21	44
Dogs’ access to cattle feed (pasture)	Yes	28	58
	No	20	42
Disposal of afterbirth or aborted foetus or dead animals	Yes	24	50
	No	24	50
Rodent control	Yes	46	96
	No	2	4

### Attitudes

Twenty-five participants (52.1%) had a positive attitude score regarding neosporosis-related risk factors. Many of the farmers demonstrated appropriate attitudes towards the key aspects of disease control, including maintaining good hygiene and sanitary conditions for cattle housing (*n* = 47/48), implementing correct biosecurity and husbandry measures to prevent and control the spread of neosporosis (*n* = 41/48), and maintaining accurate farm records (*n* = 45/48) (Table 4).

**TABLE 4:** Attitudes of South African commercial dairy farmers (*n* = 48) towards neosporosis-related risk factors.

Attitudes	Response	Frequency (*n*)	Percentage (%)
Appropriate attitude towards hygiene and sanitary conditions of cattle housing	Yes	47	98
	No	1	2
Correct implementation of measures to prevent introduction of neosporosis through maintaining a closed herd	Yes	34	71
	No	14	29
Correct implementation of measures to prevent introduction of disease through testing	Yes	7	15
	No	41	85
Correct implementation of measures to prevent introduction of disease through quarantine	Yes	9	19
	No	39	81
Correct attitude towards biosecurity and husbandry measures to control the spread of neosporosis	Yes	41	85
	No	7	15
Appropriate attitudes towards investigation of causes of reproductive failure	Yes	11	23
	No	37	77
Correct attitude towards farm record keeping	Yes	45	94
	No	3	6

### Perceptions and observations

Few farmers perceived that their cattle did not have an abortion problem (*n* = 6/48). The most common health issues on dairy farms in South Africa were mastitis (*n* = 17/48) and lameness (*n* = 10/48), while the leading reasons for culling animals were related to fertility issues (*n* = 33/48) and udder issues (*n* = 27/48) (Table 5). From the interviews, it was observed that most farmers were less concerned about animal health issues, but more worried about the increased cost of milk production. Key challenges cited by farmers as the major constraints facing the industry included electricity cuts, increasing feed costs, drought and labour shortages.

**TABLE 5:** Perceptions of South African commercial dairy farmers (*n* = 48) towards neosporosis-related health issues and other animal health issues.

Perceptions	Response	Frequency (*n*)	Percentage (%)
Abortions were an issue	Yes	42	88
	No	6	13
Ranking of the most important reasons for culling	Fertility issues	33	69
	Udder disorders	9	19
	Feet disorders	2	4
	Metabolic diseases	1	2
	Respiratory diseases	1	2
	Geriatric	1	2
	Poor milk production	1	2
Ranking of the second most important reasons for culling	Udder disorders	27	56
	Feet disorders	9	19
	Fertility issues	9	19
	Metabolic diseases	1	2
	Poor milk production	1	2
	Not sure	1	2
Main health problems in herds	Mastitis	17	35
	Lameness	8	17
	Neonatal diarrhoea	7	15
	Parasitic disease	7	15
	Bovine respiratory disease	5	10
	Reproductive diseases	3	6
	Metabolic disease	1	2
Second most important health problems in herds	Lameness	10	21
	Parasitic disease	8	17
	Mastitis	7	15
	Neonatal diarrhoea	6	13
	Reproductive diseases	6	13
	Metabolic disease	5	10
	Bovine respiratory disease	4	8
	Traumatic reticuloperitonitis	2	4
Ranking of the important causes of reproductive loss	Not sure	37	77
	Brucellosis	2	4
	Mycotoxicosis	2	4
	Chlamydiosis	2	4
	Multiple causes	2	4
	Infectious bovine rhinotracheitis	1	2
	Bovine ephemeral fever	1	2
	Plant toxicosis	1	2

### Predictors of positive epidemiological knowledge, practices and attitudes

There was no significant association between the various variables and the positive epidemiological score (Table 6), suggesting that none of these variables are predictors of commercial dairy farmers’ positive epidemiological knowledge of bovine neosporosis. However, significant association (*p* < 0.05) was found between some predictors and the positive practice and positive attitude scores. The type of feed (*p* = 0.009), disposal of afterbirth (*p* = 0.000) and management of animal records (*p* = 0.007) were associated with positive practice score (Table 6), while the level of hygiene (*p* = 0.004), biosecurity (*p* = 0.029), presence of wildlife (*p* = 0.022), presence of dogs (*p* = 0.038) and management of animal records were associated with positive attitude score (Table 7).

**TABLE 6:** Factors associated with positive practice scores regarding neosporosis among commercial dairy farmers in South Africa: results of exact multivariable logistic regression model.

Variable and level	Odds ratio (OR)	95% CI OR	*p*-value
**Region**			
Southern and coastal	1†	-	-
Central and Highveld	3.7	0.3–262.8	0.600
Northern and arid	8.9	0.6–inf	0.056
**Number of cattle**			
< 100	1†	-	-
101–500	10.4	0.5–874.9	0.109
> 500	41.7	1.4–5237.3	0.015
**Total mixed ration**			
No	1†	-	-
Yes	9.3	1.2–inf	0.027

CI OR, confidence interval odds ratio.

† , Reference level.

**TABLE 7:** Factors associated with positive attitude score regarding neosporosis among commercial dairy farmers in South Africa: results of exact multivariable logistic regression model.

Variable and level	Odds ratio (OR)	95% CI OR	*p*-value
**Region**			
Southern and coastal	1†	-	-
Central and Highveld	1.4	0.3–6.6	0.740
Northern and arid	1.5	0.1–22.5	1.000
**Presence of wildlife**			
No	1†	-	-
Yes	0.1	0.002–0.960	0.027

CI OR, confidence interval odds ratio.

† , Reference level.

### Factors associated with positive scores

Twenty-four variables, with *p* < 0.25 in the univariate analysis, were considered for the multivariable analysis. The final multivariable logistic regression model (Table 6 and Table 7) identified three variables significantly associated with the odds of positive practice and positive attitude scores. Farms with herds larger than 500 animals were 41.7 times more likely (95% confidence interval [CI]: 1.5–5237.3) to have a positive practice score. Farms that used a total mixed ration were more likely to report a positive practice score. Additionally, farms that reported the presence of wildlife were less likely to have a positive attitude score. None of the factors assessed were found to be associated with a positive knowledge score.

## Discussion

Only a small proportion of dairy farmers (20.8%) had prior knowledge of neosporosis, suggesting limited awareness of the disease and that it is not considered as a significant health or economic problem. This may be partly because neosporosis is not listed as a disease of concern by the Standard Dairy Agency in South Africa, which bases its animal health guidelines on the World Organisation for Animal Health (WOAH) Terrestrial Animal Health Code (World Organisation for Animal Health 2024). This lack of knowledge among farmers could hinder efforts to control and prevent the infection at the farm level. This issue may be exacerbated by the fact that most farmers (77.1%) said they did not follow up on the causes of abortions, even though abortion is the main clinical sign of bovine neosporosis (Almería & López-Gatius 2013).

Although most dairy farmers (95.8%) had no knowledge of the disease, the majority (77%) had a positive practice score. This suggests that farmers were implementing measures for the prevention and control of bovine neosporosis, albeit unintentionally, as some of these biosecurity measures are universal and form part of the holistic approach to control infectious diseases in animals (Maunsell & Donovan 2008). Specific biosecurity measures most relevant to neosporosis include testing and quarantining replacement animals to curb vertical transmission, which accounts for 90% of cases (Sanderson 2009). The study revealed that some farmers focused on reducing vertical transmission by testing replacement animals (14.6%) or quarantining them (18.8%). Fewer farmers took steps to reduce horizontal transmission by preventing dogs from being on the farm (10.4%). However, on farms that had dogs, many (43.8%) prevented dogs from accessing afterbirths and aborted foetuses or to feed (41.7%), both measures aimed at reducing horizontal transmission.

Most of the farmers (52.1%) had a positive attitude score regarding the correct implementation of biosecurity, biocontainment and disease risk management practices for bovine neosporosis, generally answering favourably to most of these related questions. Such positive attitudes may reflect that these farms were commercially operated on privately owned land with sufficient resources to support disease prevention, consistent with findings that farmers with privately owned land implement more biosecurity measures (Msimang et al. 2022).

The study found that 12.5% of farmers reported having no abortion problems. This could be because of the fact that 14.6% used handwritten logbooks and 6.3% kept no records, resulting in inaccurate records and reliance on memory. This may have influenced the perception that they did not have abortion issues. In contrast, farmers who used computer software (79.1%) for record keeping generally had more accurate records. Traditional manual record keeping is often erroneous, cumbersome and time-consuming, whereas computerised systems offer more advantages for dairy herd management (Rajkumar, Xavier & Anil 2008; Sánchez et al. 2020). Farmers reported the most common animal health issues as mastitis (35.4%), lameness (16.7%), neonatal diarrhoea (14.6%) and parasitic disease (14.6%). These findings align with other studies that also rank mastitis, lameness and reproductive issues as the top health concerns in dairy cattle (Wells, Ott & Seitzinger 1998). Farmers reported that the most important reasons for culling animals were fertility issues (68.8%), udder disorders (18.8%) and feet disorders (4.2%). These findings are consistent with other research that identifies claw disorders, udder disorders and fertility problems as some of the most common reasons for culling cattle (Kulkarni et al. 2023; Rilanto et al. 2020).

Three variables were significantly associated with positive practice and attitude scores. Farms with herds larger than 500 animals and those using a total mixed ration were more likely to report positive practice scores. Larger farms are typically better structured and equipped to implement good biosecurity and management practices, which may translate to higher practice scores. This aligns with studies in South Africa and Egypt that reported lower *N. caninum* seroprevalence on larger farms, attributed to better biosecurity, husbandry and management practices (Gaber et al. 2021; Tagwireyi et al. 2024). These more commercial, intensive farms routinely use total mixed rations, which are accompanied by stringent biosecurity measures that reduce the risk of feed contamination with dog faeces containing *N. caninum* oocysts. In contrast, farms that reported the presence of wildlife were less likely to have a positive attitude score. Wildlife on or near dairy farms is a documented risk factor for *N. caninum* infection, and other studies have shown that wildlife can serve as reservoirs of infection and may be perceived as greater risk, complicating disease management and negatively influencing farmers’ attitudes (Hanisch-Kirkbride, Riley & Gore 2013; Minicucci et al. 2025; Samkange et al. 2023).

A limitation of the study was the small sample size, which could have skewed the results and limited their representativeness. Commercial dairy farmers, those affiliated to farmers’ organisations and veterinarians were overrepresented, while small-scale farmers, with limited access to veterinary services, were largely excluded. These farmers are much more likely to have poorer biosecurity, weaker husbandry practices and potentially greater problems with neosporosis. Another limitation was the limited geographical coverage as only farmers from seven of the nine milk-producing provinces were surveyed.

## Conclusion

This study found that commercial dairy farmers in South Africa had limited knowledge of neosporosis. While most demonstrated the right attitudes and implemented appropriate practices related to disease prevention, these efforts were generally aimed at other diseases or focused on broad biosecurity measures. There is a need to raise awareness and to promote the testing of animals for *N. caninum*, particularly in cases of abortion and the management of dogs on farms. These measures should be integrated into a broader strategy for effective disease prevention and control programmes. This KAP study has provided valuable insights that can help address existing knowledge gaps and improve disease management of dairy farms in the country.
